# Acute liver failure associated with diffuse large B-cell lymphoma: an autopsy case report

**DOI:** 10.1007/s12328-019-01091-6

**Published:** 2020-01-09

**Authors:** Kimitoshi Kubo, Noriko Kimura, Katsuhiro Mabe, Soichiro Matsuda, Momoko Tsuda, Mototsugu Kato

**Affiliations:** 1 Department of Gastroenterology, National Hospital Organization Hakodate National Hospital, 18-16 Kawahara-cho, Hokkaido, 041-8512 Japan; 2 Department of Pathology, National Hospital Organization Hakodate National Hospital, Hakodate, Japan

**Keywords:** Acute liver failure, Malignant lymphoma, Diffuse large B-cell lymphoma, Multiple hypodense splenic lesions

## Abstract

Acute liver failure (ALF) associated with malignant infiltration of the liver is rare and its pathological and radiologic features remain poorly described. An 87-year-old man was admitted to our hospital for anorexia for several days, high-grade fever from the previous day, and liver dysfunction but suddenly died on day 3 of hospitalization due to ventricular fibrillation. The patient was diagnosed at autopsy with malignant diffuse large B-cell lymphoma. To the best of our knowledge, this report represents a valuable addition to the ALF literature describing a case of ALF associated with diffuse large B-cell lymphoma diagnosed at autopsy.

## Introduction

Acute liver failure (ALF) is defined as the presence of encephalopathy and coagulopathy (INR > 1.5) in the absence of pre-existing liver disease, with an illness of < 26 weeks’ duration [1] and its most common causes include acetaminophen overdose, idiosyncratic drug reactions, and viral hepatitis [2]. ALF associated with malignant infiltration of the liver is rare and its pathological and radiologic features remain largely unclear. We herein report a case of ALF associated with diffuse large B-cell lymphoma diagnosed at autopsy.

## Case report

**Fig. 1:**
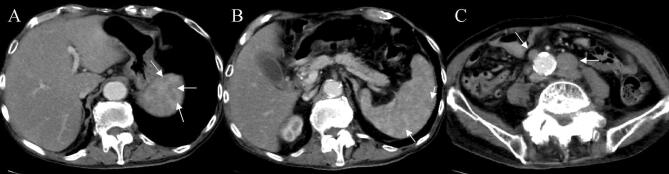
A CT examination revealed no distinct nodules or masses in the liver, but mild splenomegaly, multiple hypodense splenic lesions (**a**, **b**), and para-aortic lymph-node swelling (**c**)

**Fig. 2:**
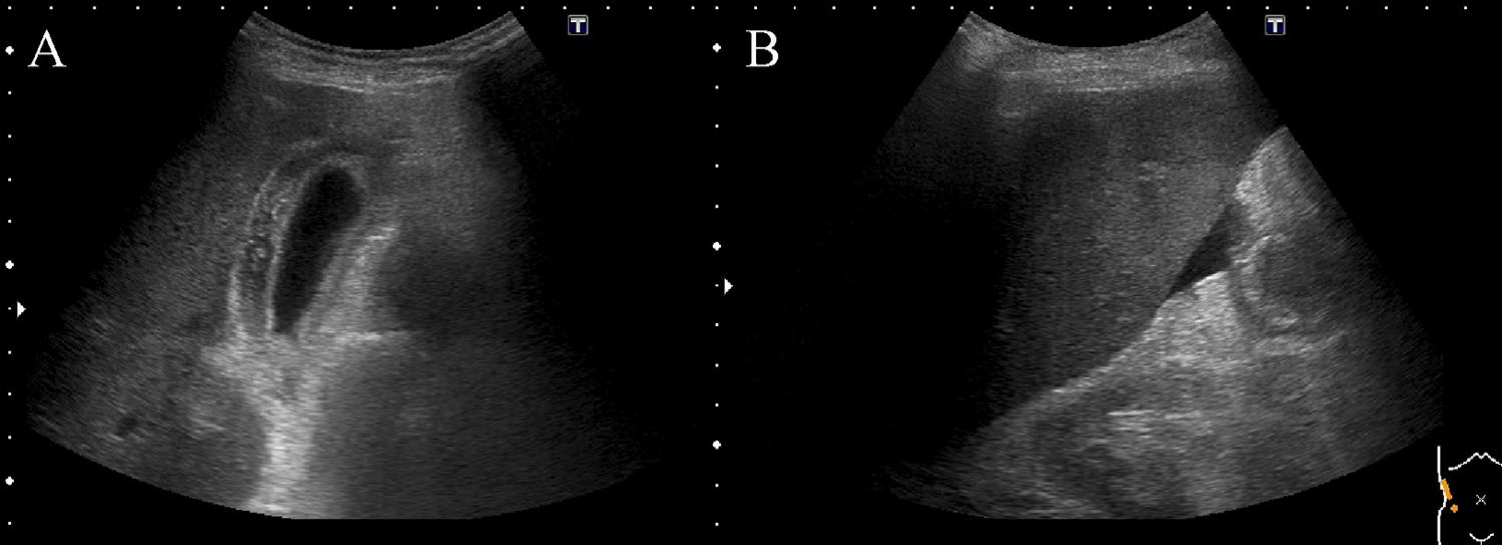
An US examination revealed thickening of the gallbladder wall (**a**) and ascites (**b**)

**Fig. 3:**
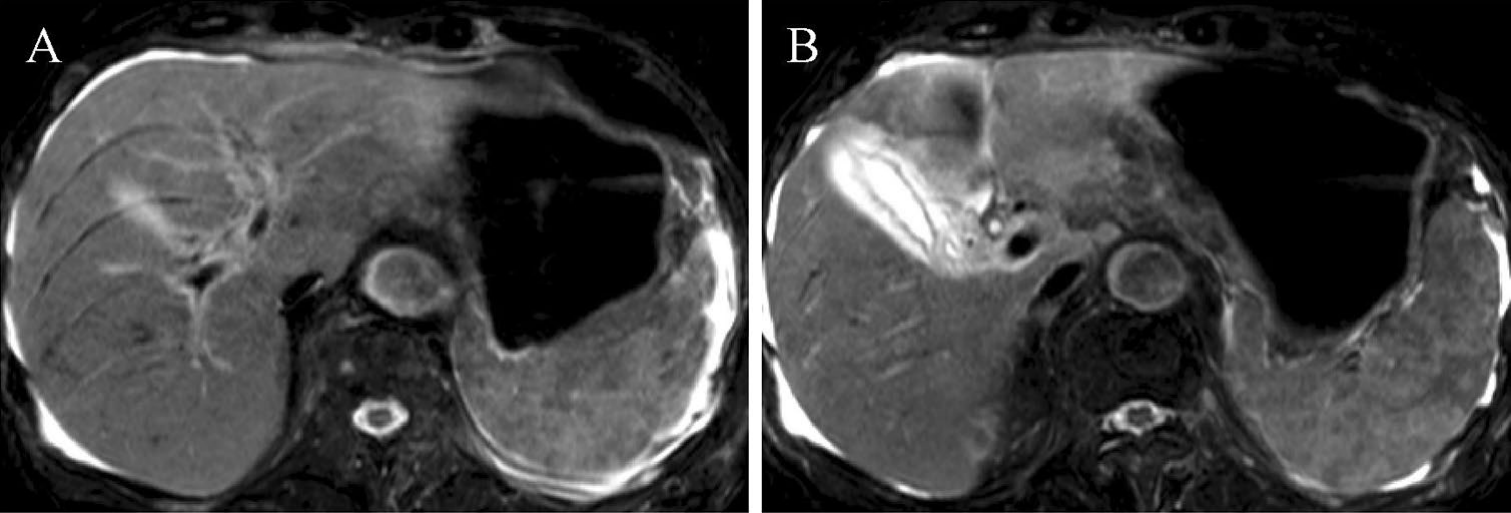
An MRI examination revealed thickening of the gallbladder wall (**a**) and ascites (**b**)

An 87-year-old man was admitted to our hospital for anorexia for several days, high-grade fever from the previous day, and liver dysfunction. Of note, he had a history of hypertension, diabetes mellitus (DM), and angina. Physical examination findings included: clear consciousness; height, 163 cm; weight, 48 kg; blood pressure, 66/40 mmHg; heart rate, 75/min; respiratory rate, 22/min; oxygen saturation of peripheral artery, 96%; and body temperature, 38.1 °C. He had no surface lymphadenopathy. Laboratory findings included: white blood cell (WBC) count, 4.2 × 10^9^/L; hemoglobin, 9.6 g/dL; platelet count, 106 × 10^9^/L; lactate dehydrogenase (LDH), 1662 IU/L; aspartate aminotransferase (AST), 6562 IU/L; alanine aminotransferase (ALT), 1407 IU/L; alkaline phosphatase (ALP), 509 IU/L; γ-glutamyl transpeptidase (γ-GTP), 130 IU/L; total bilirubin, 2.7 mg/dL; prothrombin time (PT), 20.3 s; international normalized ratio (INR), 1.73; blood urea nitrogen (BUN), 34.4 mg/dL; and creatinine, 1.4 mg/dL. Serologic tests were all negative for hepatitis A, B, C, and E. Computed tomography (CT) performed at admission revealed no distinct nodules or masses in the liver, but mild splenomegaly, multiple hypodense splenic lesions, and para-aortic lymph node swelling (Fig. 1a–c). Antibiotics were administered intravenously for suspected acute cholangitis and sepsis, but the fever persisted. Abdominal ultrasonography (US) and magnetic resonance imaging (MRI) performed 2 days later newly depicted gallbladder wall thickening and ascites (Figs. 2, 3). On the same night, the patient presented with grade II hepatic encephalopathy, with blood tests showing a decrease in platelet count and worsening liver function suggestive of “acute type” ALF with hepatic coma and disseminated intravascular coagulation (DIC). While consideration was being given to treating acute liver failure, the patient suddenly developed ventricular fibrillation and died despite immediate resuscitation measures on day 3 of hospitalization.

**Fig. 4:**
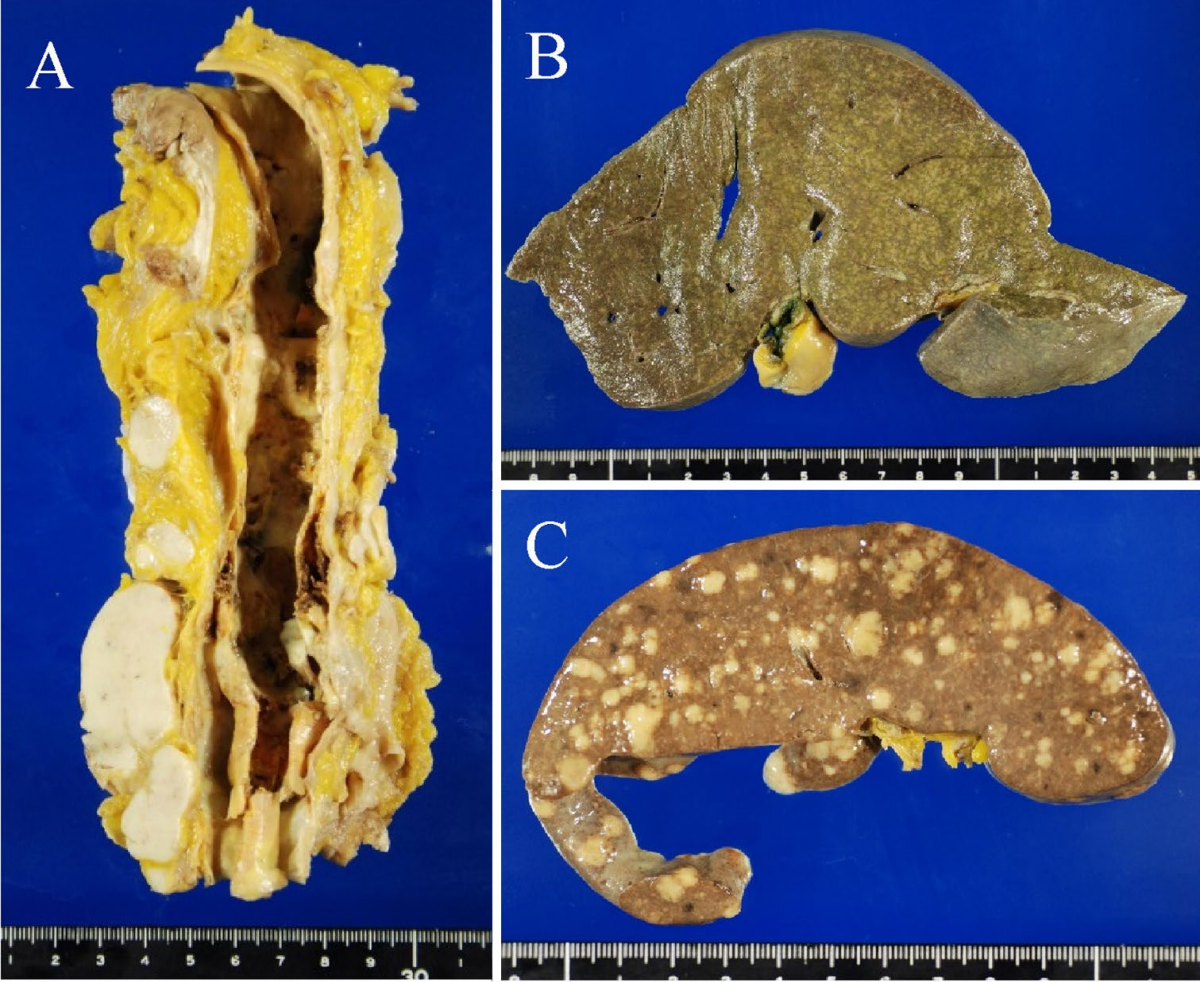
An autopsy examination revealed **a** swelling of lymph nodes in the para-abdominal aorta, **b** the spleen with multiple whitish nodules, and **c** the liver showing an irregular geographical morphology

**Fig. 5:**
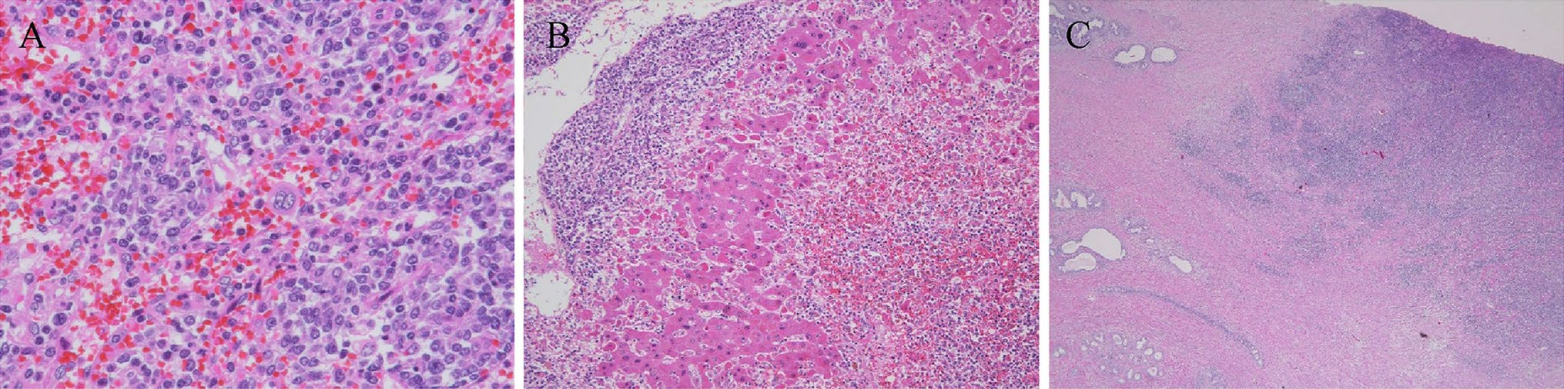
A histopathologic examination revealed **a** multiple whitish nodules in the spleen infiltrated by tumor cells, **b** the lymphoma cells mainly infiltrating the portal areas of the liver and **c** the lymphoma cells infiltrating the prostate

**Fig. 6:**
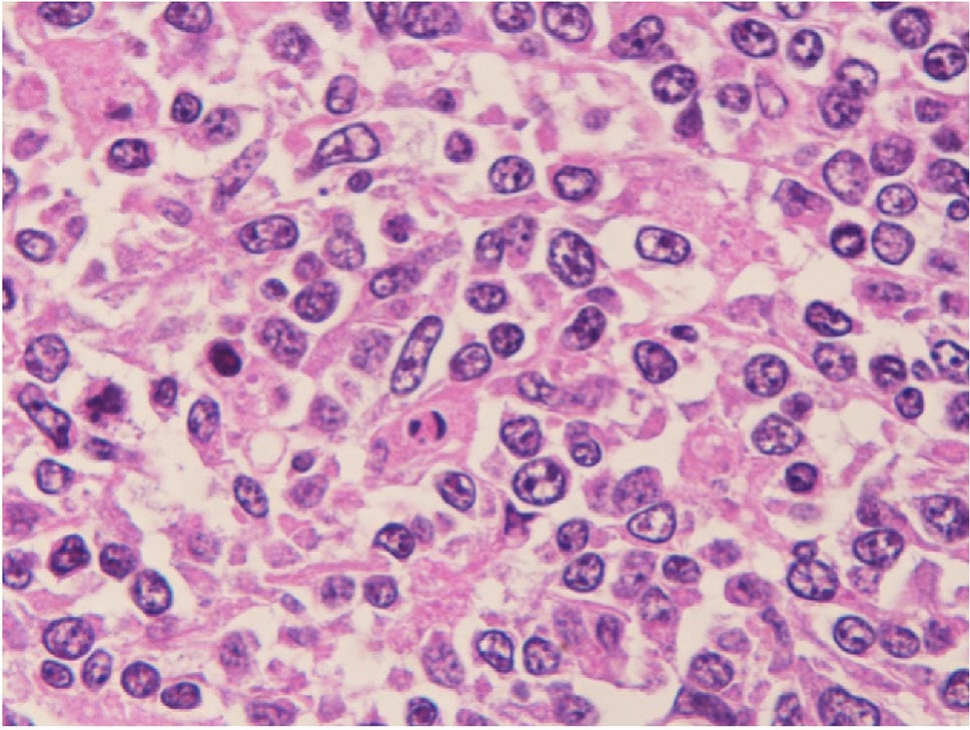
A histopathologic examination of the lymph nodes revealed medium-sized irregular nuclei and small nucleoli in the tumor cells with a high nuclear cytoplasmic ratio, showing diffuse proliferation characterized by the presence of large numbers of small T-cells

**Fig. 7:**
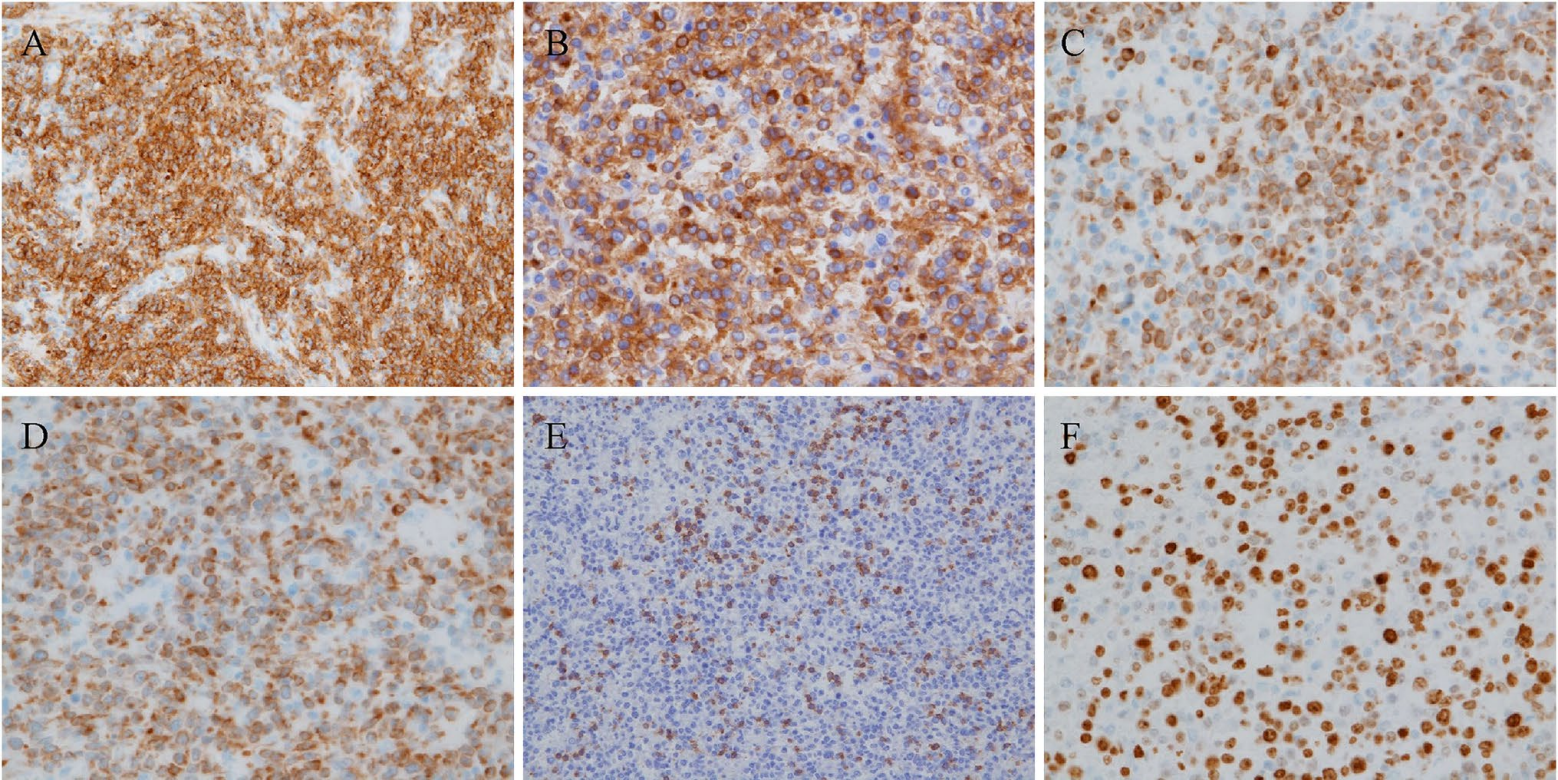
An immunohistochemistry staining of the lymph nodes revealed that the tumor cells were positive for CD20 (**a**), CD79a (**b**), bcl-2 (**c**), and MUM1 (**d**), and negative for CD3 (**e**), with an Ki-67 LI of 16% (**f**)

Given the rapid clinical course, an autopsy was performed in this patient with suspected ALF likely due to drug-induced liver injury, autoimmune hepatitis, hepatitis of indeterminate etiology, circulatory disturbance, metabolic disease, or malignant cells including malignant lymphoma. This led to the patient being diagnosed with malignant, diffuse large B-cell lymphoma, characterized by multiple lymph-node, as well as multi-organ (i.e., spleen, liver, lung, and prostate) involvement: (1) general swelling of lymph nodes in the para-abdominal aorta (Fig. 4a), peripancreatic, peribronchial, pulmonary hilar, subclavicular regions, as well as in the pelvis, especially around the iliac arteries, measuring up to 60 mm in diameter; (2) presence of multiple whitish splenic nodules (Fig. 4b) with the entire spleen shown to be microscopically infiltrated by tumor cells (Fig. 5a); (3) irregular geographical morphology of the liver (Fig. 4c) with the lymphoma cells mainly infiltrating the portal areas (Fig. 5b); (4) involvement of the right lobe of the lung; (5) involvement of both lobes of the prostate consequent to outer connective tissue involvement (Fig. 5c). Microscopically, the tumor cells in the lymph nodes were shown to have medium-sized irregular nuclei and small nucleoli with a high nuclear cytoplasmic ratio, thus showing diffuse proliferation characterized by the presence of large numbers of small T-cells (Fig. 6). Immunohistochemistry revealed the tumor cells to be positive for CD20 (Fig. 7a), CD79a (Fig. 7b), bcl-2 (Fig. 7c), and MUM1 (Fig. 7d), but negative for CD3 (Fig. 7e), CD10, bcl-6, and cyclin D1, with a Ki-67 labeling index (Ki-67 LI) of 16% (Fig. 7f). The causes of death were thus determined as (1) DIC and (2) ALF associated with diffuse large B-cell lymphoma.

## Discussion

Our case has two important clinical implications. First, diffuse large B-cell lymphoma may present as ALF. ALF associated with malignant infiltration of the liver is rare and its pathological and radiologic features remain poorly described.

Defined as the presence of encephalopathy and coagulopathy (INR > 1.5) in the absence of pre-existing liver disease, ALF is reported to last for < 26 weeks [1], most commonly due to acetaminophen overdose, idiosyncratic drug reactions, and viral hepatitis in Europe and the United States [2]. According to the Japanese diagnostic criteria, ALF is diagnosed in patients with a prothrombin time equivalent to INR of 1.5 or more due to liver injures sustained within 8 weeks of onset of disease symptoms [3], most commonly due to viral hepatitis and circulatory disturbances [4]. To date, the incidence of ALF secondary to hematological malignancies is reported to be only 0.47% (9/1910) in a multicenter study [1] and 0.44% (18/4020) in a large single-center study [5], with the overall mortality from ALF due to malignant infiltration of the liver being 85% and 94% and the median time to death following admission being 12 days (range 6–28) and 6 days (range 1–51), respectively. Recently, Nakao et al. reported the incidence of ALF due to infiltration of malignant cells as 1.81% (29/1603) in a nationwide survey of ALF conducted in Japan [4].

Of note, ALF due to diffuse large B-cell lymphoma is reported to account for only 0.26% (5/1910) [1]; to date, only ten cases of ALF due to diffuse large B-cell lymphoma have been reported (Table 1) [6–15], with the time to death following admission in six reported cases being 5 days [12, 13], 8 days [7, 9], 14 days [14], and 19 days [6], in contrast to only 3 days in the present case. It has been reported that the deaths were caused by multi-organ failure involving cardiovascular collapse, acute lung injury, and renal failure, in addition to hepatic failure [5]. Recently, Sakae et al. reported an autopsy report demonstrating the spread of malignant lymphoma to multiple lymph nodes, as well as the spleen, liver, lung, epicardium, adrenal glands, prostate, and bone marrow [12].

**Table 1:** Cases reported to date of acute liver failure associated with diffuse large B-cell lymphoma

No	References	Year	Age	Sex	Imaging findings	Diagnostic modality	Treatment	Prognosis
1	[6]	1998	75	M	CT: hepatosplenomegaly	Liver/spleen necropsy	PSL	Dead (day 19)
2	[7]	2003	48	M	(–)	BM/liver biopsy	Chemotherapy	Dead (day 8)
3	[8]	2008	53	F	CT: splenomegaly	Liver biopsy	Chemotherapy	Alive (as of 1 year)
4	[9]	2014	62	M	CT: hepatosplenomegaly and lymphadenopathy	BM/liver biopsy	PSL	Dead ( day 8)
5	[10]	2014	71	M	US: fatty liver, mild splenomegaly, and mild distended gallbladder CT: multiple hypodense splenic lesions, a confluent non-compressing circum-aortic mass, multiple para-aortic and pelvic lymph nodes, and bilateral pulmonary nodules	Lymph node biopsy	Chemotherapy	Dead
6	[11]	2016	60	M	CT: hepatosplenomegaly	BM biopsy	Chemotherapy	Alive
7	[12]	2016	66	M	US: splenomegaly and a hyperechoic mass in the spleen CT: hepatosplenomegaly and a mass with fluid–fluid levels in the spleen	Autopsy	(–)	Dead (day 5)
8	[13]	2017	57	M	CT: hepatic steatosis, splenomegaly with multiple splenic infarcts, and lymphadenopathy	Liver biopsy	Chemotherapy	Dead (day 5)
9	[14]	2018	55	F	US, CT, and MRCP: diffuse hepatomegaly	BM biopsy	PSL	Dead (day 14)
10	[15]	2019	33	F	US: atrophied potato liver with massive ascites CT: atrophy of the liver with a mixed density area, splenomegaly, and lymphadenopathy	Gastric biopsy	PSL Chemotherapy	Alive
11	Our case	2019	87	M	CT: mild splenomegaly, multiple hypodense splenic lesions, and para-aortic lymph nodes swelling US and MRCP: thickening of the gallbladder wall and ascites	Autopsy	(–)	Dead (day 3)

BM, bone marrow; CT, computed tomography; MRCP, magnetic resonance cholangiopancreatography; PSL, prednisolone; US, ultrasonography

In the present case, an autopsy examination revealed the spread of malignant lymphoma to multiple lymph nodes, as well as to the spleen, liver, lung, and prostate, suggesting that the malignant lymphoma had already infiltrated multiple organs by the time of onset of ALF. On day 3 of hospitalization, with his hyperthermia shown to be persistent, the patient developed tachycardia at 97 bpm and tachypnea at 27/min suggesting systemic inflammatory response syndrome (SIRS). Based on the negative blood culture results obtained later, DIC due to ALF associated with diffuse large B-cell lymphoma was thought to be the cause of death in our patient. As such, this report is a valuable addition to the ALF literature describing a case of ALF associated with diffuse large B-cell lymphoma diagnosed at autopsy.

While there have been a few case reports showing that long-term survival could be achieved with chemotherapy in patients with ALF [8, 11, 15] and that the diagnosis of ALF is facilitated by liver biopsy, bone marrow biopsy, and gastric biopsy (Table 1), Rich et al. highlighted the importance of early tissue sampling through liver biopsy [1], suggesting that early diagnosis by biopsy and treatment are crucial for survival in patients with ALF. However, liver biopsy should be carefully considered in ALF patients depending on their risk of complications, which may have been increased with coagulopathy and thrombocytopenia.

The second implication of our case is that CT revealed no distinct nodules or masses in the liver but multiple hypodense lesions in the spleen. It has been reported that radiographic studies are typically notable for the absence of large mass lesions and reveal only a nodular liver in some cases that may be described as pseudocirrhosis, given that fulminant presentations are commonly associated with diffuse intra-sinusoidal infiltration [1]. Additionally, hepatosplenomegaly and lymphadenopathy may also be seen on CT [1, 5], and the CT findings are shown to be similar in the ten cases reported to date (Table 1). While two cases of multiple hypodense splenic lesions have been reported in ALF associated with malignant lymphoma, no pathological examination has been performed in these cases [10, 16]. Of note, a pathological examination in our case clearly revealed that the multiple hypodense splenic lesions detected were metastatic lesions of the primary malignant lymphoma. This finding, including the presence of lymphadenopathy on CT, may prove helpful in predicting the presence of ALF associated with malignant lymphoma.

In conclusion, diffuse large B-cell lymphoma presented as ALF. ALF associated with malignant lymphoma should be suspected in patients with ALF in the absence of distinct nodules or masses in the liver but in the presence of multiple hypodense lesions in the spleen or lymphadenopathy on CT.
